# Technical advance in targeted NGS analysis enables identification of lung cancer risk-associated low frequency TP53, PIK3CA, and BRAF mutations in airway epithelial cells

**DOI:** 10.1186/s12885-019-6313-x

**Published:** 2019-11-11

**Authors:** Daniel J. Craig, Thomas Morrison, Sadik A. Khuder, Erin L. Crawford, Leihong Wu, Joshua Xu, Thomas M. Blomquist, James C. Willey

**Affiliations:** 1 Department of Medicine, The University of Toledo College of Medicine, 3000 Arlington Avenue, Toledo, OH 43614 USA; 2 Accugenomics, Inc, 1410 Commonwealth Dr #105, Wilmington, NC 28403 USA; 3 National Center for Toxicological Research, U.S. Food & Drug Administration, Jefferson, AR USA; 4 Department of Pathology, The University of Toledo College of Medicine, 3000 Arlington Avenue, Toledo, OH 43614 USA

**Keywords:** Biomarker, Low-frequency variant detection, Next generation sequencing, Lung Cancer

## Abstract

**Background:**

Standardized Nucleic Acid Quantification for SEQuencing (SNAQ-SEQ) is a novel method that utilizes synthetic DNA internal standards spiked into each sample prior to next generation sequencing (NGS) library preparation. This method was applied to analysis of normal appearing airway epithelial cells (AEC) obtained by bronchoscopy in an effort to define a somatic mutation field effect associated with lung cancer risk. There is a need for biomarkers that reliably detect those at highest lung cancer risk, thereby enabling more effective screening by annual low dose CT. The purpose of this study was to test the hypothesis that lung cancer risk is characterized by increased prevalence of low variant allele frequency (VAF) somatic mutations in lung cancer driver genes in AEC.

**Methods:**

Synthetic DNA internal standards (IS) were prepared for 11 lung cancer driver genes and mixed with each AEC genomic (g) DNA specimen prior to competitive multiplex PCR amplicon NGS library preparation. A custom Perl script was developed to separate IS reads and respective specimen gDNA reads from each target into separate files for parallel variant frequency analysis. This approach identified nucleotide-specific sequencing error and enabled reliable detection of specimen mutations with VAF as low as 5 × 10^− 4^ (0.05%). This method was applied in a retrospective case-control study of AEC specimens collected by bronchoscopic brush biopsy from the normal airways of 19 subjects, including eleven lung cancer cases and eight non-cancer controls, and the association of lung cancer risk with AEC driver gene mutations was tested.

**Results:**

TP53 mutations with 0.05–1.0% VAF were more prevalent (*p* < 0.05) and also enriched for tobacco smoke and age-associated mutation signatures in normal AEC from lung cancer cases compared to non-cancer controls matched for smoking and age. Further, PIK3CA and BRAF mutations in this VAF range were identified in AEC from cases but not controls.

**Conclusions:**

Application of SNAQ-SEQ to measure mutations in the 0.05–1.0% VAF range enabled identification of an AEC somatic mutation field of injury associated with lung cancer risk. A biomarker comprising TP53, PIK3CA, and BRAF somatic mutations may better stratify individuals for optimal lung cancer screening and prevention outcomes.

## Background

Lung cancer is the leading cause of cancer-related death in men and women, and cigarette smoking is the most significant preventable risk factor [1]. Despite widespread smoking cessation initiatives, due to past and continued cigarette use, as well as the lack of effective treatment for advanced disease, lung cancer will continue to be the deadliest cancer for decades to come [2].

The primary strategies to reduce lung cancer death are prevention through reduction in exposure to tobacco products and screening of high-risk subjects by annual low-dose CT (LDCT) scan to diagnose lung cancer when it is in early stage and curable [3]. Annual LDCT screening significantly reduces lung cancer mortality [4]. However, there is large inter-individual variation in lung cancer risk among those currently recommended for screening according to demographic criteria [5]. Overall, lung cancer incidence is low (< 10%) among those who currently meet screening criteria [5, 6]. Thus, there is a need for an effective biomarker that will more accurately stratify individuals according to lung cancer risk, improve specificity, and thereby reduce cost and harms related to LDCT screening. One approach toward this goal is to characterize differences in the prevalence and characteristics of somatic cell genetic damage in histologically normal airway epithelium of lung cancer cases compared to controls matched for smoking and age [7–9]. This idea is supported by the presence of extensive morphologic and molecular changes in the airway epithelium of lung tissue from heavy smokers, including large chromosomal changes and point mutations, and higher prevalence of these changes in subjects with lung cancer than in non-cancer subjects matched for smoking and age [7, 10, 11].

Inter-individual variation in airway epithelial cell (AEC) somatic mutation prevalence may be due to variation in the relative contribution of a) random DNA replicative errors during stem cell division and subsequent tissue regeneration, b) environmental (e.g., smoking, radon, asbestos) factors that increase risk for replicative errors due to DNA damage, and c) hereditary germline DNA variants associated with sub-optimal protection of DNA from damage and/or damage repair [12, 13]. Thus, it is reasonable to hypothesize that prevalence of somatic mutations among certain genes in AEC will represent a summation biomarker for the interactive effects of stochastic replicative errors, hereditary risk variants, and cigarette smoke exposure on lung cancer risk.

Advances in next generation sequencing (NGS) technology markedly increase the ability to measure somatic mutations in AEC and other tissues. In a recent study, targeted NGS capable of measuring mutations with variant allele frequency (VAF) > 1.0% was used to assess driver gene somatic mutations in lung cancer tissue and adjacent matched normal tissue from a group of subjects [14]. A large number of mutations known to be drivers for lung cancer were identified in non-cancer lung tissues in close proximity to each cancer. As such, measurement of mutations with VAF > 1% may support development of biomarkers for early diagnosis and/or genetic characterization of a prevalent lung cancer. However, the clone prevalence diminished proportional to the distance from the cancer site, with very few mutants in the normal airway of the lung not affected by the cancer or in nasal epithelium. As such, this approach did not support development of a non-invasive test for future incidental lung cancer risk.

Untested so far is the hypothesis that lung cancer predisposition may be measured as increased prevalence and/or a characteristic spectrum of low variant frequency (VAF < 1.0%) mutations in AEC. Testing this approach presented a challenge due to limited sensitivity and reliability of current prevalent NGS analysis methods. Working together with the FDA-sponsored Sequencing Quality Control Consortium (SEQC), we identified actionable best practices for mutation detection in clinical applications using NGS technologies [15]. We then implemented these best practices while applying the novel Standardized Nucleic Acid Quantification SEQuencing (SNAQ-SEQ) method in a retrospective case-control study. According to SNAQ-SEQ, we mixed synthetic DNA internal standards (IS) into each AEC gDNA specimen prior to NGS library preparation. The IS enabled identification of nucleotide site-specific technical error and clear identification of low VAF mutation signal in samples relative to noise (technical artifacts arising from sequencing error). Thus, synthetic IS provided a reproducible measure of technical error in test samples [16] as is routinely done to ensure quality-control in other key molecular diagnostic testing methods, including liquid and gas chromatography and mass spectroscopy [17, 18] and the FDA-approved Roche COBAS® qPCR tests. We previously reported that position-specific and mutation-specific technical error observed in IS DNA is highly correlated with technical error in respective sample DNA measured in the same sequencing library [16]. As implemented here, SNAQ-SEQ was able to measure variants at VAF as low as 5 × 10^− 4^ (0.05%). We also demonstrated the value of including IS in targeted NGS for both RNA-Seq and mutation analysis [11, 19, 20].

In this study, target genes were chosen for analysis based on high prior likelihood of being mutated in lung cancer as reported by The Cancer Genome Atlas (TCGA) study, including some considered to be drivers of malignancy [21].

## Methods

### Study cohort enrollment and characterization

For this retrospective case-control study, we used AEC specimens collected from nineteen subjects, including eleven smokers with lung cancer (CA-SMK), five smokers without cancer (NC-SMK) matched for age and smoking history, and three non-smokers without cancer (NC-NON) (Table 1). Subjects were enrolled into research trials at the University of Toledo Medical Center (UTMC) between 2000 and 2018. Each subject included in this research study provided written informed consent under protocols approved by the University of Toledo Institutional Review Board. Clinical characteristics, including lung cancer diagnosis, smoking history, and demographic information were obtained from the medical record. Lung cancer histology was reviewed and confirmed by an independent pathologist certified in anatomical and clinical pathology.

**Table 1:** Patient Demographics

Sample #	Cancer Status	Pack Years	Sex	Age Range	Smoking Status	Diagnosis
946	CA	45	F	50–59	Former	NSCLC-SQ
167	CA	50	F	60–69	Unknown	NSCLC
947	CA	45	M	60–69	Former	SCLC
146	CA	46.5	F	60–69	Former	NSCLC
887	CA	28	F	70–79	Current	NSCLC-AD
885	CA	90	M	70–79	Current	SCLC
940	CA	60	M	70–79	Former	NSCLC-AD
191	CA	NA*	M	70–79	Current	NSCLC-SQ
147	CA	75	M	70–79	Former	SCLC
128	CA	40	F	50–59	Current	NSCLC
923	CA	15	M	70–79	Former	NSCLC
210	NC	34	M	40–49	Current	Noncancer
886	NC	0	F	40–49	Never	Noncancer
952	NC	30	M	50–59	Former	Noncancer
157	NC	100	M	60–69	Unknown	Noncancer
943	NC	0	F	60–69	Never	Noncancer
956	NC	20	M	60–69	Current	Noncancer
884	NC	54	M	70–79	Former	Noncancer
883	NC	0	M	80–89	Never	Noncancer

*Not available: The exact pack year smoking history for this patient was not recorded. However, it was recorded that the patient was an active 2 PPD smoker at time of lung cancer diagnosis at age 75 and had advanced stage COPD, thus there is compelling circumstantial evidence for large smoking history

### Definition of non-Cancer subjects (Additional file 1: table S1)

Subjects were defined as non-cancer based on negative chest CT (no nodules or masses reported by Radiologist) at time of sample collection, or specific benign pathological diagnosis of CT abnormality followed by confirmation of no lung cancer 2 or more years after sample collection. When possible, subjects’ medical records were reviewed at least yearly to determine lung cancer status. Indeed, there was one subject (subject #128) that was diagnosed with lung cancer 11 years after sample collection and this subject was treated as a cancer in this report.

### Specimen acquisition

AEC were obtained via bronchoscopic brush biopsy of normal appearing airway epithelium at the time of a diagnostic procedure done according to standard of care indication as previously described in detail [22]. For patients with a lung cancer diagnosis, sampling of AEC was from the main bronchus of the lung not involved with cancer. Specimens were immediately placed in cold saline and processed within one hour of collection.

### DNA extraction and quantification

Genomic DNA (gDNA) was extracted from approximately 500,000 AEC per subject using a FlexiGene DNA kit (Qiagen, Hilden, Germany) according to manufacturer protocol and quantified using competitive polymerase chain reaction (PCR) amplification of a well-characterized genomic locus in the Secretoglobin, family 1A, member 1 gene as described previously [23].

### Target selection

Twelve loci in seven gene regions recently reported by The Cancer Genome Atlas (TCGA) project to be the most commonly mutated in non-small cell lung cancer were selected as targets [21]. The targeted regions, specified according to Human Genome Organization (HUGO) names with exon numbers and abbreviations provided in parentheses, included B-Raf proto-oncogene exon 15 (BRAF_15), epidermal growth factor receptor exons 18–21 (EGFR_18, EGFR_19, EGFR_20, EGFR_21), erb-b2 receptor tyrosine kinase 2 (ERBB2), KRAS proto-oncogene exon 2 (KRAS_2), notch receptor 1 exon 26 (NOTCH1_26), phosphatidylinositol-4,5-bisphosphate 3-kinase catalytic subunit alpha exon 10 (PIK3CA_10), and tumor protein p53 exons 5–7 (TP53_5, TP53_6, TP53_7).

### SNAQ-SEQ method

#### Reagent synthesis

Primers and synthetic internal standard mixtures were prepared for SNAQ-SEQ at Accugenomics, Inc. (Wilmington, NC) for each of the selected targets (Additional file 2: Table S2). Primers for all targets except for NOTCH1_26 performed efficiently in multiplex and downstream library preparation. As such, data are reported for the remaining 11 targets.

#### Synthetic internal standard mixture preparation

Competitive synthetic DNA internal standard (IS) molecules for TCGA targets described above were designed with known dinucleotide substitution mutations relative to target analyte native template (NT) every 50 bases. This enabled separation of NT and IS reads during post-sequencing data processing of either PCR amplicon libraries used in this study, or of random fragment hybrid capture libraries in other ongoing studies not reported here. IS were cloned into plasmids and selected as pure clonal isolates using Sanger sequencing confirmation to verify the final sequence. This additional purification step was taken to select clones free of any potential errors introduced by synthesis. Based on prior studies, due to the high fidelity of endogenous E.coli polymerase, the frequency of variants in the cloned IS can be expected to be between 10^− 7^ to 10^− 8^ [24]—well below the desired limit of detection for this study. Each cloned plasmid was linearized, quantified by digital droplet PCR, then combined in an equal genome copy balance. An internal standard mixture (ISM) containing equal concentrations (per genome copy) of each linearized target analyte IS molecule was prepared. We previously reported that technically-derived base substitution errors occur at the same rate in synthetic IS as in the respective target sequence within gDNA test samples during the combined library preparation and sequencing steps. Therefore, each IS controls for target-specific site and regional differences in base substitution error rate [16].

#### Multiplex competitive PCR amplicon libraries

In order to amplify each target in a sample and maximize opportunity to detect low frequency variants, a multiplex competitive PCR amplicon library was prepared for each AEC DNA sample. Conditions were optimized to minimize technical error during PCR, including use of Q5 HotStart High Fidelity DNA Polymerase with a reported error frequency of 10^− 6^ (New England Biolabs, Ipswich, MA) and minimization of PCR cycles in each round.

#### Round 1: competitive multiplex PCR

Twelve target-specific primers with universal tails were synthesized by Life Technologies (Carlsbad, CA). Individual primer solutions for each target were created by adding TE buffer (10 mM Tris-Cl, pH 7.4, 0.1 mM EDTA) to the lyophilized primers to make a 100 μM stock. A 2.5 μM multiplex primer mixture was prepared by mixing 5 μL of each 100 μM forward and reverse primer stock solution and bringing the final volume to 200 μL with TE buffer.

For each subject, an aliquot of AEC DNA was combined with equal genome copies of ISM to control for nucleotide-specific substitution error occurring during library preparation and/or sequencing. Reactions containing at least 50,000 genome equivalents of both sample and IS in a mixture, 6 μL 5X Q5 Buffer (New England Biolabs, Ipswich, MA), 0.6 μL 10 mM dNTP (Promega, Madison, WI), 3 μL 2.5 μM multiplex primer mixture, 1.5 μL 2% w/v bovine serum albumin (New England Biolabs, Ipswich, MA), 0.3 μL Q5 HotStart High Fidelity DNA Polymerase (New England Biolabs, Ipswich, MA, Ipswich, MA), and molecular-grade water to a final reaction volume of 30 μL were prepared.

Each competitive multiplex reaction mixture was amplified in a 7500 Fast Real-Time PCR System (Applied Biosystems, Foster City, CA) for a total of 20 cycles under modified gradient PCR conditions: 95 °C/2 min (Q5 HotStart DNA Polymerase activation); 20 cycles of 94 °C/10 s (denaturation), 70 °C/10 s, 68 °C/10 s, 66 °C/10 s, 64 °C/10 s, 62 °C/10 s, (annealing), and 72 °C/30 s (extension); a final extension 72 °C/2 min extension to ensure complete extension of all products. PCR products were column-purified using QIAquick PCR Purification Kit (Qiagen, Hilden, Germany) according to manufacturer protocol.

#### Round 2: Singleplex PCR

Following multiplex amplification, a second round of 12 parallel singleplex PCR reactions using primers for each individual target at a final concentration of 500 nM were performed to ensure robust amplification of product for primers with lower efficiency in multiplex. High fidelity Q5 Hot Start Polymerase and other PCR reagents were used as described above.

Singleplex reactions were amplified in a 7500 Fast Real-Time PCR System (Applied Biosystems, Foster City, CA) for 15 cycles using the following conditions: 95 °C/2 min (Q5 polymerase activation); 15 cycles of 94 °C/10 s (denaturation), 65 °C/20 s, (annealing), and 72 °C/30 s (extension); a final extension 72 °C/2 min extension was performed to ensure complete extension of all products. Each singleplex PCR product was checked for quality and quantity with an Agilent 2100 Bioanalyzer using DNA Chips with DNA 1000 Kit reagents according to manufacturer protocol (Agilent Technologies, Deutschland GmbH, Waldbronn, Germany). Sample-specific singleplex reactions then were (a) mixed in equimolar amounts to ensure an equal balance of target reads among sequencing read counts and (b) column-purified using QIAquick PCR Purification Kit (Qiagen, Hilden, Germany) according to manufacturer protocol.

#### Round 3: addition of sample-specific barcodes

The column-purified mixture of singleplex reactions from each patient sample was labeled using a unique set of dual-indexed barcode primers to reduce likelihood of false-indexing/barcoding a sequencing read [25]. A pair of fusion primers containing the barcode sequences and Illumina priming sites were designed with 1) their 3′-end complementary to the universal sequence tails added during the initial multiplex and singleplex reactions, 2) 5′ to that a 10-nucleotide index/barcode sequence, and [3] 5′ to that, an Illumina Read 1 or Read 2 priming site. The final concentration of the barcode primers in each reaction was 500 nM. PCR conditions were identical to those described for singleplex reactions except the cycle number was reduced to 10.

PCR products were checked for quality and quantity with an Agilent 2100 Bioanalyzer using DNA Chips with DNA 1000 Kit reagents according to manufacturer protocol and diluted 100-fold with molecular grade water for input into final sequencing adapter PCR.

#### Round 4: addition of sequencing adapters

Individual diluted barcoded samples were labeled with an Illumina platform-specific adapter using a second set of fusion primers designed with their 3′-end complimentary to the Illumina Read 1 or Read 2 priming sites and 5′ Illumina sequencing adapter using the same PCR conditions used in Round 3.

#### Sample pooling

Following Round 4, each uniquely barcoded sample was quantified on an Agilent 2100 Bioanalyzer as described above. The samples then were mixed in equimolar ratios to optimize the percentage of sequencing reads that each library would eventually receive; in most cases 1∶1 was used.

#### Product purification and sequencing

The combined sequencing library was purified using gel electrophoresis on a 2% w/v agarose gel. The resultant product band was then cut out, separating it from unwanted heterodimers, extracted using a QIAquick Gel Extraction Kit (Qiagen, Hilden, Germany), and eluted in 50 μL elution buffer. The purified sequencing library was sent to the University of Michigan Genomics core facility for Next Generation Sequencing on an Illumina NextSeq 550 sequencing instrument.

### Analysis of NGS data

FASTQ data files generated by the University of Michigan Genomics core facility were processed using a custom Perl script to separate the internal standard (IS) and native template (NT) reads into separate NT and IS files (Additional file 7: File 1), followed by parallel analysis using the Qiagen CLC Genomics Workbench 12 software suite for quality-trimming, alignment, and variant calling (Additional file 7: File 1). Primer sequences, internal standard dinucleotide positions plus their 5′ and 3′ bases, and known single nucleotide polymorphism (SNP) positions were excluded from variant analysis.

#### Variant calling

Variants were called based on NT signal significantly above the background error measured in IS for the respective mutation type at each respective position. Significance was determined using contingency table chi-square analysis of each individual variant type at each nucleotide position, as previously described for identifying rare variants in pooled samples [26]. To maximize stringency of test for signal above noise, a variant was called if the proportion of variant reads to wild-type reads in the specimen was significantly higher than the proportion of variant reads to wild-type reads at the same site in the IS mixed with the respective specimen, and also higher than the proportion observed in IS mixed with each of the other 18 specimens. Thus, each variant in a specimen was considered a true positive (*p* < 0.05) only if the proportion of variant reads to wild-type reads was significantly higher in the specimen relative to each of the 19 IS replicates. A Bonferroni correction for false discovery was used based on the number of nucleotides assessed (760 bp) and the number of substitution mutations possible at each nucleotide position. Further, to avoid potential analytical variation from stochastic sampling, only mutations with significant signal above IS noise, and with VAF > 0.05% were called.

#### Variant annotation and hotspot analysis

Called variants were characterized for pathogenicity using publicly-available databases including dbSNP, COSMIC, and FASMIC. Identification of known oncogenic hotspots and generation of corresponding figures were assessed using the cBioPortal for Cancer Genomics developed at Memorial Sloan Kettering (MSK) Cancer Center [27].

#### Statistical analysis

Calling of variants based on contingency table chi-square analysis of each individual variant type at each nucleotide position was performed using R: A Language and Environment for Statistical Computing (http://www.R-project.org/). Assessment of hotspot enrichment for called variants was performed using Kruskal-Wallis test using a chi-square distribution. Mutation prevalence based on type of mutation and target was assessed using Kruskal-Wallis test with Nemenyi test for multiple comparisons (Additional file 8: File 2).

## Results

### Measurement of low frequency mutations in non-cancer airway epithelium

**Fig. 1:**
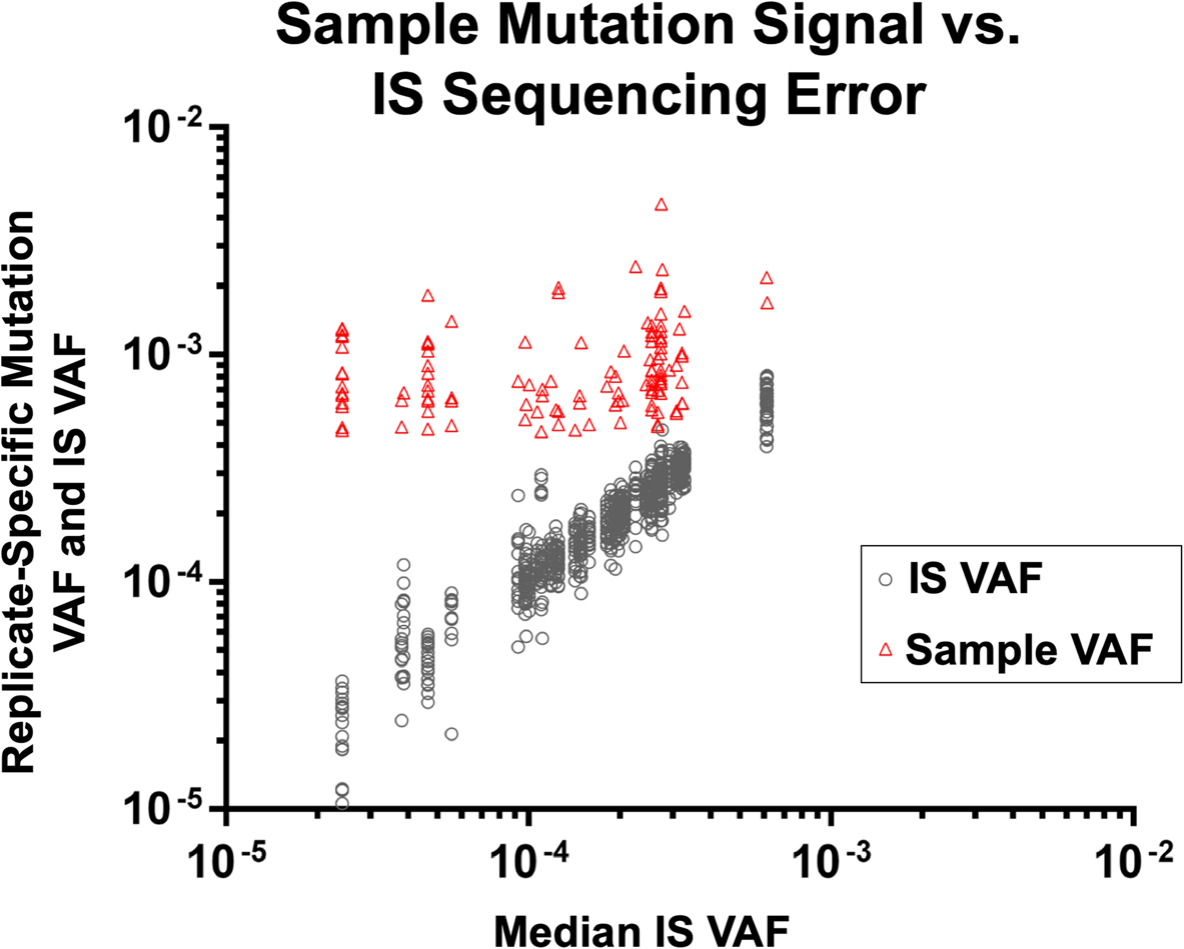
Mutations identified in patient specimens. Sample mutation signal versus IS sequencing error. Variant allele frequency (VAF) of sample mutations (red triangle) relative to VAF of corresponding nucleotide-specific error variants in 19 IS replicates (black circle). VAF = site specific variant allele reads/total allele reads

In this study of 11 driver gene target regions in AEC specimens from normal airways of 19 subjects, there were 129 called variants with VAF ranging from 5 × 10^− 4^ (0.05%) to 4.6 × 10^− 3^ (0.46%) (Additional file 3: Table S3). As described in the Methods section, a VAF minimum threshold of 0.05% was used to minimize risk of false discovery due to stochastic sampling. Among the 129 called variants, the relationship between sample mutation signal (Mutation VAF) and background technical error (noise) (IS VAF) for the respective variant at the same site is presented in Fig. 1. For each sample mutation VAF, there is displayed the IS VAF for 19 IS. These represent the VAF for the IS mixed with the sample that contained the mutation as well as the VAF for each of the IS mixed with the other 18 samples. These 19 independent IS replicate values represent the variation around the IS VAF (error) measurement within an experiment. As is evident, the inter-replicate variation in IS VAF values increases with decreasing IS VAF, consistent with effects of the Poisson distribution on stochastic sampling as previously reported [16] (Fig. 1, Additional file 3: Table S3). These effects of Poisson distribution presented challenges for statistical analysis of significance for observed sample mutations (Additional file 3: Table S3) that we addressed through use of a non-parametric contingency table approach.

### Characteristics of sequencing error in the targeted regions

As is evident from Fig. 1 and Additional file 3: Table S3, at sites within the targeted regions for which a sample variant was called the maximum technical error (Median IS VAF across replicates) observed was 0.06%. This error rate is lower than the median error rate observed for non-targeted sequencing on a comparable Illumina platform [28] [29].

### Prevalence of low frequency mutations in AEC

Mutation prevalence was calculated as called mutations per nucleotide positions assessed for each target. The number of nucleotides assessed for each target varied somewhat based on region spanned by primers and number of dinucleotide sites blocked from analysis due to modification in IS to enable separation of IS reads from NT reads. Among all 19 subjects, the average mutation prevalence, across the summary of targeted DNA regions (760 bp) in each subject (mutations/bp/subject) was 8.9 × 10^− 3^. (Table 2). This AEC mutation prevalence value is much higher than reported for methods that only detect mutants with relatively high variant frequency (VAF > 1%) [14], or that are more sensitive but non-targeted [30]. However, it is consistent with our previous analysis of AEC using a highly sensitive PCR-based method [31, 32].

**Table 2:** Target- and cohort-specific mutation prevalence

Target	CA-SMK	NC-SMK	NC-NON	NC-TOT	Average (All Subjects)
BRAF_15	6.7 × 10^− 3^	0	0	0	3.9 × 10^− 3^
EGFR_18	0	0	0	0	0
EGFR_19	0	0	0	0	0
EGFR_20	3.9 × 10^−2^	3.4 × 10^− 2^	4.5 × 10^− 2^	3.8 × 10^− 2^	3.8 × 10^− 2^
EGFR_21	1.7 × 10^− 3^	0	0	0	9.9 × 10^− 4^
ERBB2	1.1 × 10^− 2^	1.4 × 10^− 2^	1.4 × 10^− 2^	1.4 × 10^− 2^	1.2 × 10^− 2^
KRAS_2	0	0	0	0	0
PIK3CA_10	4.2 × 10^− 3^	0	0	0	2.4 × 10^− 3^
TP53_5	2.2 × 10^− 2^	4.7 × 10^− 3^	0	2.9 × 10^− 3^	1.4 × 10^− 2^
TP53_6	2.2 × 10^− 2^	0	3.1 × 10^− 3^	1.2 × 10^− 3^	1.3 × 10^− 2^
TP53_7	1.3 × 10^− 2^	2.9 × 10^− 3^	0	1.8 × 10^− 3^	8.5 × 10^− 3^
Average (All Targets)	1.2 × 10^− 2^	4.7 × 10^− 3^	5.3 × 10^− 3^	4.9 × 10^− 3^	8.9 × 10^− 3^

Mutations were defined as substitutions with VAF (variant allele reads/total allele reads) > 5 × 10^− 4^ and significantly above IS VAF (i.e., background noise) based on contingency table analysis. Mutation prevalence was defined as mutations/target bp/subject (see Methods)

### Association of low frequency substitution mutations in TP53, PIK3CA, and BRAF with Lung Cancer

**Fig. 2:**
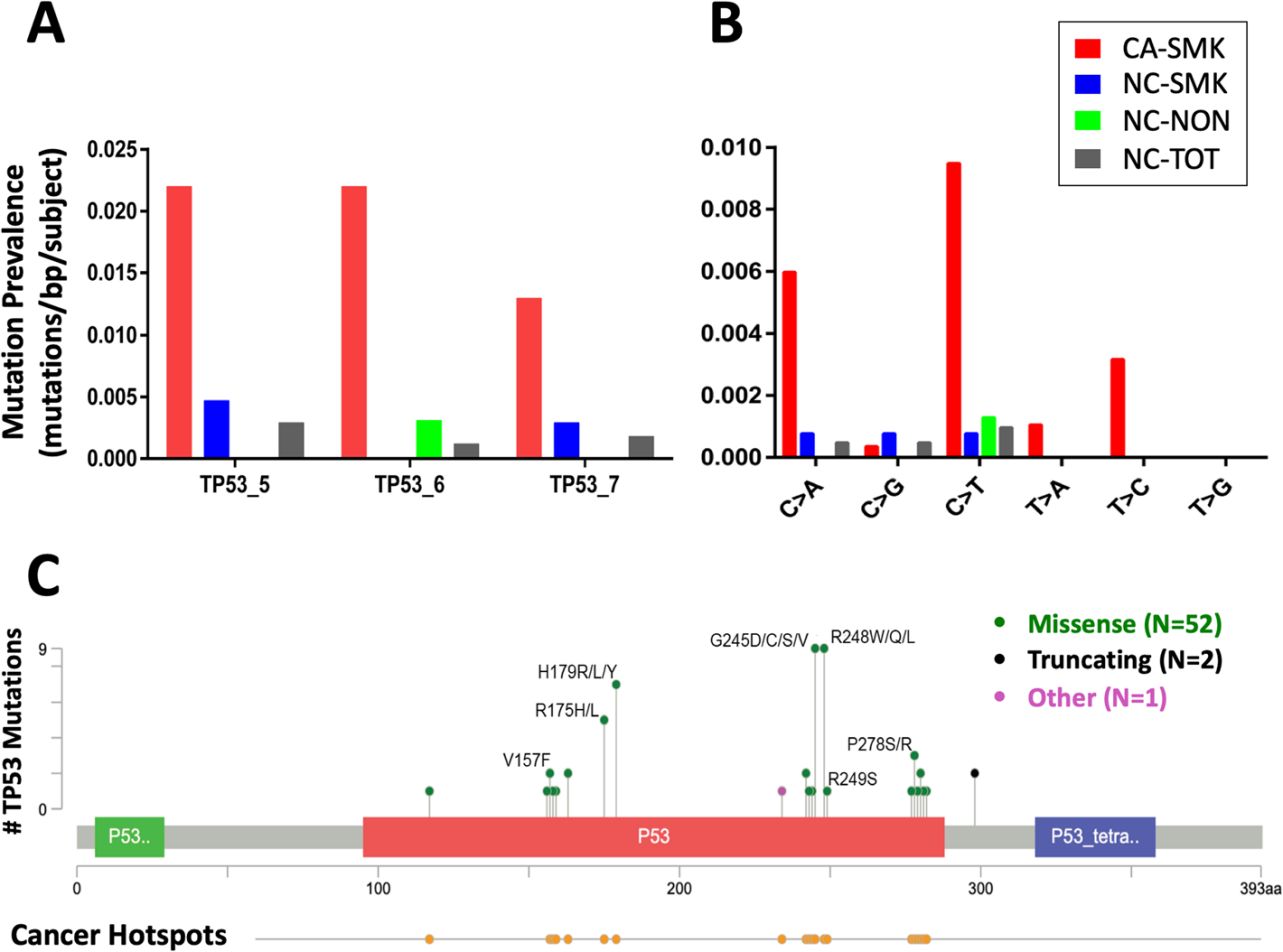
Inter-cohort comparison of TP53 mutation mean prevalence. **a** Mean mutation prevalence among subjects within each cohort in each separate TP53 exon 5, 6, or 7 (mutations/target base/subject). **b** Cohort- and substitution-specific mean mutation prevalence for the combined three TP53 exon targets. **c** Number of mutations at TP53 hotspot sites. Inset: number of mutations according to mutation type. Mutations were defined as those with VAF (variant allele reads/total allele reads) > 0.05% and significantly above IS background VAF based on contingency table analysis (see Methods). TP53 mutations in CA-SMK subjects were enriched significantly at “hotspot” lung cancer driver mutation sites (*p* = 0.002)

Among the three measured exons of TP53, the prevalence (mutations/bp/subject) of substitution mutations was 10.4-fold higher (*p* < 0.05) in AEC from CA-SMK subjects relative to NC-SMK subjects matched for smoking and age (Fig. 2a, Table 3). In addition, PIK3CA or BRAF mutations were observed in seven cancer subjects and no non-cancer subjects (Table 4). Notably, the majority of mutations in TP53 (Fig. 2c), all of the mutations in PIK3CA, and one of three mutations in BRAF (Additional file 6: Fig. S2) occurred in previously identified “hotspots” associated with biological changes that drive carcinogenesis [21, 33].

**Table 3:** Statistical analysis of target specific inter-cohort differences in mutation prevalence

Target	CA-SMK vs. NC-TOT	CA-SMK vs. NC-SMK	CA-SMK vs. NC-NON	NC-SMK vs. NC-NON
BRAF_15	0.12	0.4	0.54	1
EGFR_18	N/A	N/A	N/A	N/A
EGFR_19	N/A	N/A	N/A	N/A
EGFR_20	0.72	0.78	0.96	0.74
EGFR_21	0.39	0.76	0.83	1
ERBB2	0.35	0.73	0.8	1
KRAS_2	N/A	N/A	N/A	N/A
PIK3CA_10	0.062	0.27	0.41	1
TP53_5	0.022	0.27	0.1	0.77
TP53_6	0.0083	0.037	0.333	0.849
TP53_7	0.028	0.25	0.16	0.9
TP53_Total	0.0019	0.047	0.043	0.92

*p*-value for differences in mutation prevalence in each target across cohorts was measured with Kruskal-Wallis test and *p*-values presented were adjusted for multiple comparisons using Nemenyi test. Mutations were defined as those with VAF (variant allele reads/total allele reads) > 5 × 10^− 4^ and significantly above IS background VAF based on contingency table analysis

**Table 4:** Distribution of mutations across targets and samples

Sample	Diagnosis	Cohort	BRAF_15	EGFR_18	EGFR_19	EGFR_20	EGFR_21	ERBB2	KRAS_2	PIK3CA_10	TP53_5	TP53_6	TP53_7	Total
946	CA	SMK	1	0	0	0	0	0	0	0	0	1	0	2
167	CA	SMK	0	0	0	3	0	1	0	0	1	3	3	11
947	CA	SMK	0	0	0	2	0	1	0	1	0	0	1	5
146	CA	SMK	1	0	0	3	1	1	0	0	1	0	0	7
887	CA	SMK	0	0	0	4	0	1	0	0	1	0	1	7
885	CA	SMK	0	0	0	3	0	0	0	0	4	2	1	10
940	CA	SMK	0	0	0	0	0	0	0	1	6	6	2	15
191	CA	SMK	0	0	0	3	0	2	0	1	2	5	1	14
147	CA	SMK	1	0	0	3	0	1	0	1	4	4	0	14
128	CA	SMK	0	0	0	3	0	1	0	0	0	1	0	5
923	CA	SMK	0	0	0	1	0	1	0	0	2	4	1	9
210	NC	SMK	0	0	0	3	0	1	0	0	0	0	0	4
886	NC	NON	0	0	0	2	0	1	0	0	0	0	0	3
952	NC	SMK	0	0	0	1	0	1	0	0	1	0	0	3
157	NC	SMK	0	0	0	2	0	1	0	0	0	0	1	4
943	NC	NON	0	0	0	3	0	1	0	0	0	1	0	5
956	NC	SMK	0	0	0	3	0	1	0	0	1	0	0	5
884	NC	SMK	0	0	0	1	0	1	0	0	0	0	0	2
883	NC	NON	0	0	0	3	0	1	0	0	0	0	0	4
Total			3	0	0	43	1	17	0	4	23	27	11	129

Inter-target and inter-sample distribution of mutations. Mutations were defined as those with VAF (variant allele reads/total allele reads) > 5 × 10^−4^ (0.05%) and significantly above IS background VAF based on contingency table analysis

**Fig. 3:**
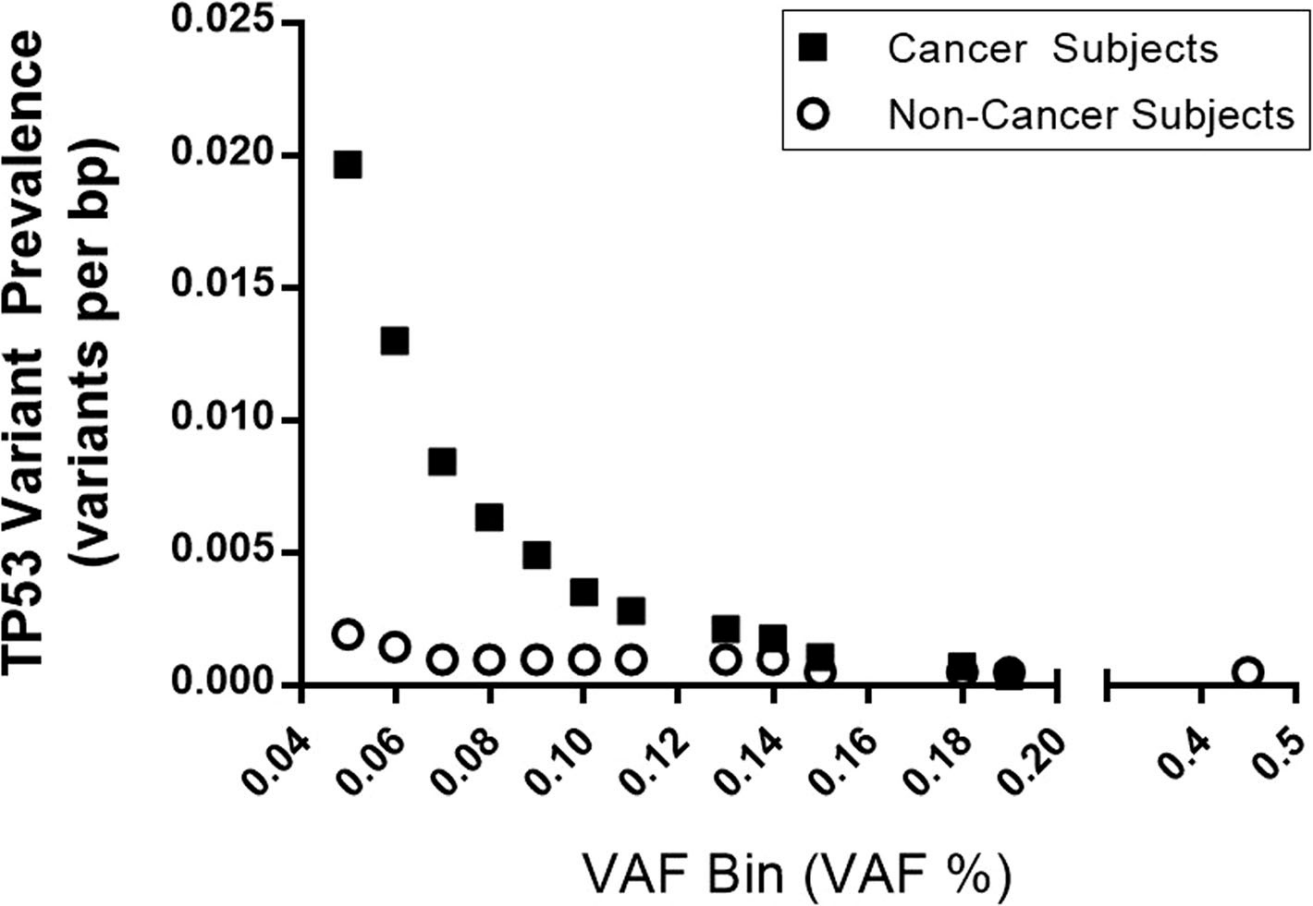
Effect of VAF cut-off on TP53 mutation prevalence detected in AEC of subjects with or without cancer. Hotspot regions in TP53 exons 5, 6, and 7 were targeted. Variants were binned according to VAF lower limit and cumulative variants in cancer (solid symbol) or non-cancer subjects (open symbol) above the indicated VAF threshold were plotted

As is evident in Fig. 3, the prevalence of AEC mutations in TP53 exons 5,6, and 7 increased at a lower VAF range and the separation between cancer and non-cancer AEC was most prominent between 0.05 and 0.1% VAF. Importantly, and consistent with prior studies of field effect in AEC [14], there were no mutations measured in AEC above 1% VAF.

**Fig. 4:**
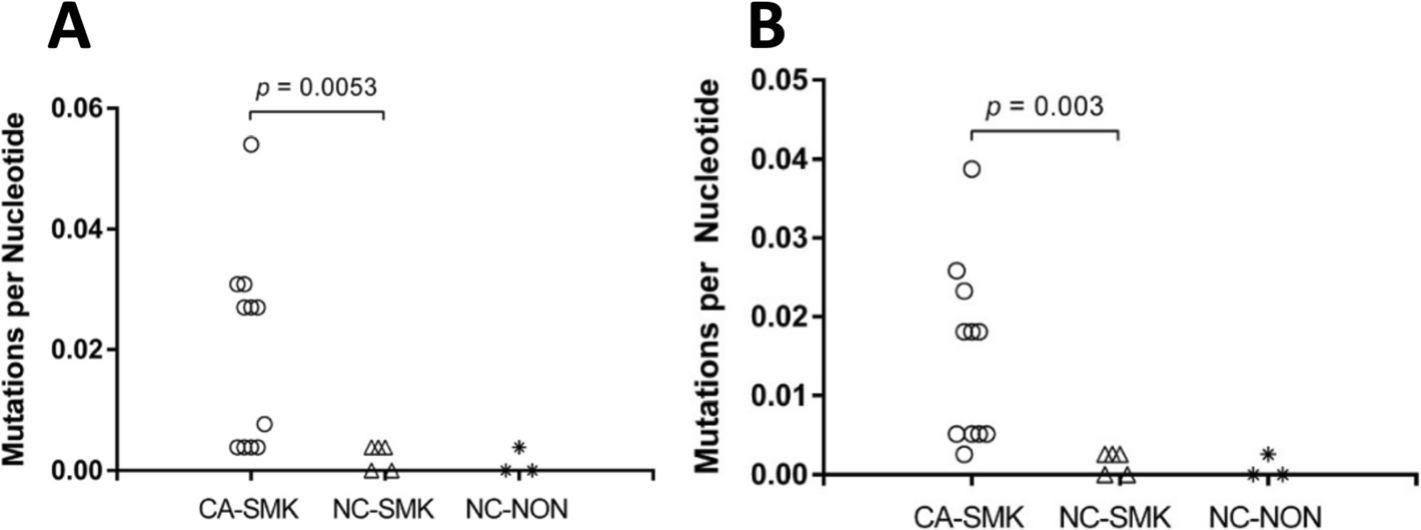
Inter-cohort comparison of subject-specific mutation prevalence. Inter-cohort comparison of subject-specific mutation prevalence (mutations/target base/subject) in (**a**) TP53 exons only or (**b**) TP53 exons, PIK3CA, and BRAF

Toward the goal of developing a biomarker that might contribute to improved determination of lung cancer risk, we assessed subject-specific inter-cohort differences in prevalence of these low frequency mutations (Fig. 4). Based on data obtained in this small retrospective case-control study, a TP53 exon mutation prevalence cut-off of 0.002 mutations/bp would have 100% specificity and 55% sensitivity (Fig. 4a). Similar discrimination was observed when TP53 exon mutations were combined with PIK3CA, and BRAF mutations (Fig. 4b).

Nearly all of the TP53 mutations in CA-SMK subjects were tobacco signature or age-related mutations (C > A, C > T, and T > C substitutions) (Fig. 2B, Table 5), closely approximating the spectrum of TP53 mutations reported for lung cancer tissues [34, 35]. The prevalence of each type of tobacco or age signature TP53 mutation was significantly higher in cancer subjects than in non-cancer subjects, including C > A (*p* = 0.002), C > T (*p* = 0.003), and T > C (*p* = 0.001) (Table 5). For example, while C to A mutations comprised 29.8% (17/57) of TP53 mutations observed in AEC from CA-SMK subjects, there was only one C to A TP53 mutation observed in all non-cancer subjects (NC-TOT) (Table 5). C > T transitions comprised 47% of TP53 mutations in lung cancer subjects in this study. Further, TP53 mutations in CA-SMK subjects were enriched significantly (*p* = 0.002) at “hotspot” lung cancer driver mutation sites (Fig. 2c) [33, 34].

**Table 5:** Inter-cohort comparison of type-specific substitution mutations across all TP53 exons

Mutation	CA-SMK^1^	NC-SMK^2^	NC-NON^3^	NC-TOT^4^
C > A	17 (2.0 × 10^−3^)*	1 (1.2 × 10^−4^)	0	1 (1.2 × 10^−4^)
C > G	1 (1.2 × 10^−4^)	1 (1.2 × 10^− 4^)	0	1 (1.2 × 10^− 4^)
C > T	27 (3.2 × 10^−3^)***	1 (1.2 × 10^− 4^)	1 (1.2 × 10^− 4^)	2 (2.4 × 10^− 4^)
T > A	3 (3.6 × 10^− 4^)	0	0	0
T > C	9 (1.1 × 10^− 4^)*	0	0	0
T > G	0	0	0	0

^1^CA-SMK; Cancer subject, present or past smoker. ^2^NC-SMK; Non-Cancer subject, present or past smoker. ^3^NC-NON; Non-Cancer subject, never smoker. ^4^NC-TOT; All Non-Cancer subjects, smokers and non-smokers

*p < 0.05; **p < 0.01; ***p < 0.005

Mutation number with prevalence in parentheses (mutations/target bp/subject) for each substitution type. Mutations were called as described in Methods section, after testing for significance of mutation VAF above background and using a VAF of 0.05% as a minimum threshold

### Lack of association of TP53 mutations with smoking history

Notably, among non-cancer subjects, smoking was not associated with higher TP53 mutation prevalence (Table 3), and this is consistent with our prior study [31]. Specifically, only half of NC-SMK subjects had even a single TP53 mutation with VAF > 0.05% and in each case, only one variant was observed. (Fig. 3, Table 4). Due to the small number of PIK3CA and BRAF mutations it was not possible to assess these for a statistically significant association with smoking.

### Characteristics of low frequency AEC mutations not associated with Lung Cancer

**Fig. 5:**
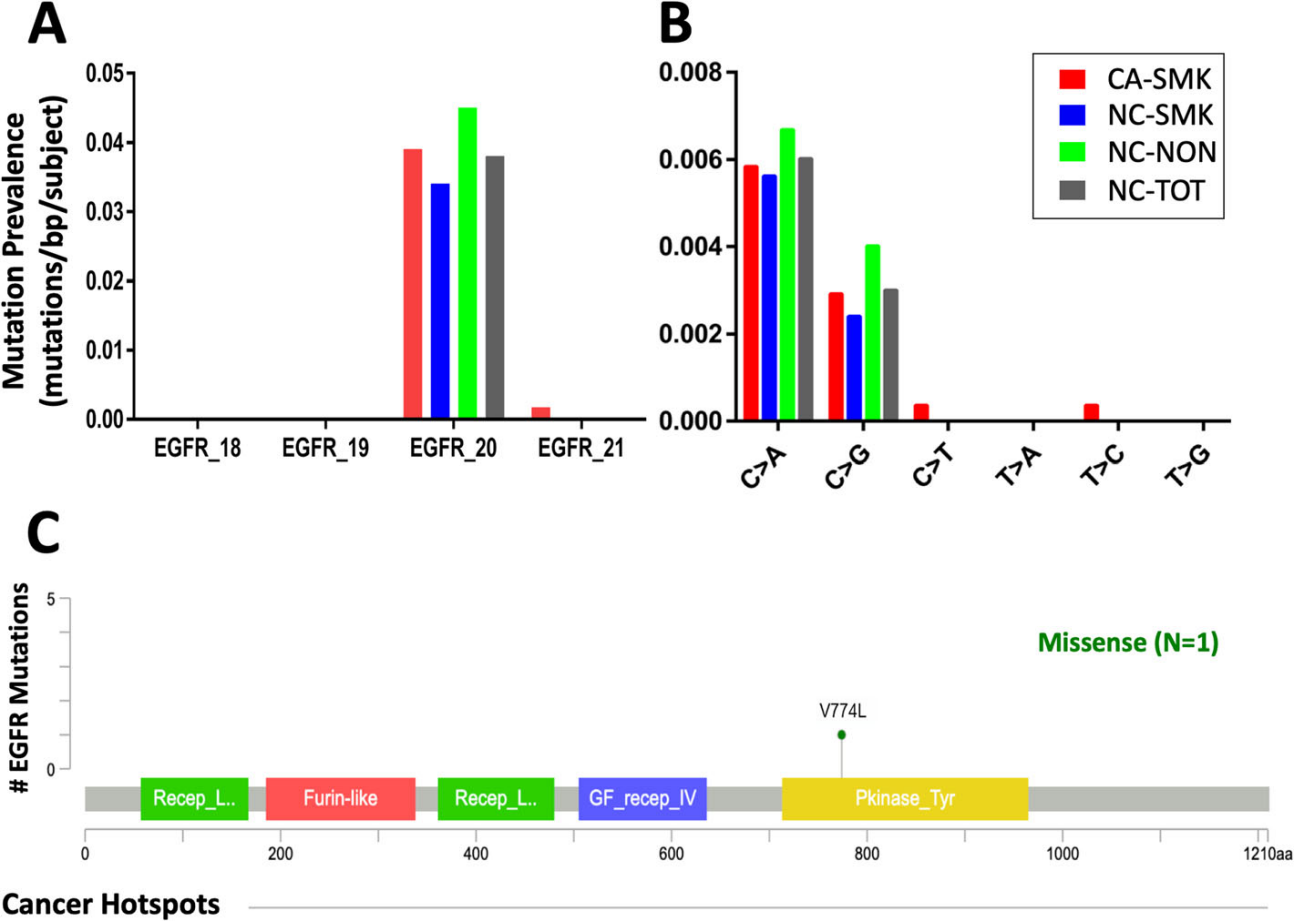
Inter-cohort comparison of EGFR mutation mean prevalence. **a** Mean mutation prevalence among subjects within each cohort in each EGFR exon [18–21], or (mutations/target base/subject). **b** Cohort- and substitution-specific mean mutation prevalence for the combined four EGFR exon targets. **c** Number of mutations at EGFR hotspot sites. Inset: number of mutations according to mutation type. Mutations were defined as those with VAF (variant allele reads/total allele reads) > 5 × 10^− 4^ (0.05%) and significantly above IS background VAF based on contingency table analysis (see Methods)

In contrast to TP53, at non-TP53 targets the mutation prevalence was not significantly different in cancer compared to non-cancer subjects (Table 3). Among the 11 targets measured, mutation count was highest in the EGFR_20 target region with a total of 43 mutations observed across all subjects (Table 4). There was no difference in EGFR_20 mutation prevalence between cancer and non-cancer (3.9 × 10^− 2^ vs 3.8 × 10^− 2^, respectively; *p* = 0.72) (Fig. 5a, Table 3), and no association between smoking and non-smoking (3.4 × 10^− 2^ vs 4.5 × 10^− 2^ respectively; *p* = 0.74). ERBB2 mutations (*N* = 17) displayed a similar spectrum to that of EGFR_20 with no age or tobacco signature mutation pattern and no difference among the cohorts. Notably, in contrast to the high fraction of C > T transitions among TP53 (29/61; 48%), only 1/43 (2.3%) EGFR_20 mutations, and 1 ERBB2 mutation was C > T (Fig. 4b, Additional file 4: Table S4). Further, the majority of the EGFR_20 mutations were synonymous and not predicted to be pathogenic (Fig. 4c).

## Discussion

### Measurement of low frequency mutations in AEC

Use of synthetic internal standards in the novel SNAQ-SEQ method to measure low frequency AEC somatic mutations revealed a lung cancer-associated TP53 mutation field effect (Figs. 3 and 4). This TP53 mutation field effect would not have been evident if less sensitive NGS methods were used, as in a recent study that employed a method with VAF lower limit of > 1% [14]. Inclusion of synthetic internal standards with confirmed reference sequence in each library sample preparation enabled qualitative and quantitative characterization of technical error for each type of variant (transition and/or transversion) at each nucleotide site in each library. This approach enabled determination of significance relative to background error for each measurement (Fig. 1) as is desirable for all diagnostic applications [36]. Use of synthetic IS, as described here for targeted NGS diagnostics, is analogous to IS applications that are now standard in liquid and gas chromatography, and mass spectrometry diagnostic applications [17, 18]. As such, use of the approach presented here for error control is highly suited for analysis of somatic mutations with VAF > 0.05% in driver gene regions. Due to practical limits on size of clinical specimens available for NGS analysis, it is reasonable to consider the specimen-determined lower limit for mutation VAF to be > 0.05% [37].

As is clear from Fig. 1 and Additional file 3: Table S3, for the targeted driver gene regions spanned in this study, the median technical error VAF measured in IS for corresponding true positive sample variants was 0.014%. This error rate is similar to that reported from other studies that employed targeted NGS on an Illumina platform to assess cancer driver gene hotspot regions and is much lower than the approximately 0.1% median error reported for non-targeted NGS on Illumina platforms [29]. These results strongly support the conclusion that targeted NGS analysis of actionable mutations with synthetic IS and without use of unique molecular indexes (UMI) has potential to improve quality control and throughput while reducing costs.

### A TP53 mutation field effect associated with lung cancer risk

The higher prevalence of low frequency TP53 hot-spot pathogenic tobacco smoke and age signature mutations in AEC of CA subjects compared with NC subjects matched for smoking and age represents a field of injury strongly associated with lung cancer risk (Figs. 2a, b, 4a, Tables 3, 5). The observed enrichment for TP53 mutations in driver mutation sites and for tobacco-smoke signatures provided another source of validation that the observed mutations are true positives. These results justify further evaluation of low frequency (i.e., VAF < 1%) TP53 hotspot mutations in AEC as a lung cancer risk biomarker. Moreover, inclusion of low frequency actionable mutations in BRAF and PIK3CA may further enhance accuracy of this proposed biomarker (Fig. 4b). If the AEC biomarker is validated in larger case/control and prospective cohort studies, it will justify studies in surrogate specimens that may be obtained through a method less invasive than bronchoscopy, such as brushing of nasal epithelium and/or collection of exhaled breath condensate. The range of prevalence for low frequency TP53 mutations in AEC among subjects in this study was similar to that previously observed using a highly sensitive PCR method that employed synthetic internal standards [31, 32].

### Higher prevalence of low frequency TP53 mutations in AEC of subjects with lung cancer

The observed higher prevalence of TP53 mutations in cancer subjects reported in this study is consistent with previously reported greater somatic cell genetic damage in histologically normal airway epithelium of lung cancer cases compared to controls matched for smoking and age [7–9]. Together, these observations support the hypothesis that lung cancer predisposition is due, in part, to hereditary and/or acquired sub-optimal protection of AEC DNA from damage associated with cigarette smoking. For example, there is large inter-individual variation in regulation of key DNA repair, antioxidant, and cell-cycle control genes in AEC [11, 20, 38–40], and the lung cancer risk test (LCRT) based on this variation has high accuracy to identify lung cancer subjects [11]. The LCRT prospective validation study is currently in progress (NCT01130285) [22]. One of the variables in the LCRT biomarker is TP53 transcript abundance, and there is a 100-fold variation in TP53 expression in AEC [11, 20]. TP53 plays a key role in upregulating DNA repair genes in response to DNA damage [41], and TP53 protein directly regulates the key nucleotide excision repair (NER) gene, ERCC5, in AEC [42]. We recently determined that germline allelic variation at rs2296147, a TP53 recognition site in the 5′-regulatory region of ERCC5, is associated with variation in allele-specific expression of ERCC5 in AEC [38–40]. Hereditary inter-individual variation in ERCC5 transcription regulation by TP53 is significant because ERCC5 is the rate-limiting enzyme in transcription-coupled NER, and mutations associated with tobacco smoke result from inefficient NER of DNA adducts arising from the binding of cigarette smoke carcinogen metabolites to the exocyclic N2-positions of guanines on the transcribed strand [41, 43]. Thus, it is reasonable to hypothesize that sub-optimal ERCC5 regulation by TP53, determined by inherited germ line variants, is an important factor responsible for higher prevalence of tobacco smoke induced hotspot mutations in the transcribed strand of TP53 among cancer subjects. Given the association of lung cancer risk with two orthogonal biomarkers measured in AEC (i.e., low frequency TP53 cigarette smoke signature somatic mutations on the one hand, and gene expression patterns on the other), a logical next step is to assess the performance of each type of biomarker in AEC from the same subjects. This will clarify whether the two biomarkers are measuring the same or independent informative risk factors and whether a composite biomarker will be more informative of risk.

### Lung cancer-associated TP53 mutations are limited to small AEC clones

The prevalence of low frequency TP53 variants is elevated in AEC of lung cancer subjects and this prevalence decreases with increasing VAF (Fig. 3). A plausible explanation for this is that as the TP53 mutation cell clone size increases, driven in part by reduced TP53 function, neo-antigens are detected and attacked by the immune-surveillance system. This explanation is consistent with a recent report that markers of immune activation are observed in pre-malignant lesions [44]. Thus, it is likely that the vast majority of TP53 driven clones are detected and removed by immune surveillance, even among individuals who have a high burden due to sub-optimal DNA protection, as described in the prior section. However, due to the high prevalence of TP53 driven clones in those at higher risk for lung cancer, there is greater risk that with reduced immune surveillance in advanced age and perhaps due to a regional sub-optimal immune surveillance deficit, a clone will escape detection, acquire additional driver mutations, and begin to invade.

### Interpretation of non-pathogenic EGFR mutations

In contrast to TP53, for EGFR the prevalence of AEC somatic mutations was not different between cancer and non-cancer subjects or smokers and non-smokers (Fig. 5a,b; Table 2, Table 3). Further, the substitution pattern (evenly distributed between C > A and C > G) is not associated with cigarette smoke exposure [35]. Moreover, evidence presented here supports the conclusion that the observed EGFR exon 20 mutations do not confer growth advantage. Specifically, in contrast to the observed non-synonymous pathogenic TP53 smoke- and age-related mutations, only one of the 43 observed EGFR_20 mutations was non-synonymous and present at a known pathogenic hotspot (Fig. 5c). A reasonable explanation, consistent with our observations in a prior study, is that clonal populations with this type of mutation (i.e., not associated with growth advantage, not associated with cigarette smoke signature) likely occurred as stochastic DNA replication errors in stem cell proliferation to generate the airway epithelium during the fetal-juvenile period [31, 32, 45].

### Potential biomarkers to guide targeted chemoprevention

Currently, there is no targeted therapy for lung cancer-associated TP53 mutations. However, there are targeted therapies for PIK3CA and BRAF driver mutations and mutations at PIK3CA or BRAF hotspots were detected in the AEC of six of the 11 lung cancer subjects and none of the non-cancer subjects (Table 4). For each subject in this study, DNA was extracted from approximately 500,000 AEC, and for each of the six subjects positive for PIK3CA or BRAF mutations, the average mutation VAF was about 10^− 3^. Thus, if clones were evenly distributed at a similar prevalence, using a prior estimation of 5 × 10^8^ AEC throughout bronchial trees of both lungs [32], we would expect a total of 10^5^ mutations in 1000 colonies per subject. Relatively non-toxic gene targeted therapies for PIK3CA and BRAF are FDA-approved or in advanced trials for some cancers. For example, *alpelisib* is currently in Phase III trials for treatment of PIK3CA driver mutations in cancers of the lung and other tissues [46], and a combination of *dabrafenib* and *trametinib* has clear efficacy in treatment of BRAF:V600E mutated non-small cell lung cancers [47]. Thus, if the PIK3CA/BRAF prevalence in AEC is validated in a larger study, it would be reasonable to consider trials in which the AEC mutation spectrum is measured before and after treatment of lung cancer subjects bearing cancers that also have the mutation. This would enable testing of the hypothesis that relatively well-tolerated gene targeted therapy could reduce the burden of AEC field of injury mutations that contribute to development of lung cancer. If the hypothesis is supported, then individuals with elevated PIK3CA/BRAF mutation prevalence in AEC could be considered for chemoprevention trials.

### Statistical analysis comments

The contingency table statistical approach employed to determine significance of observed sample mutations [26] was useful in this study and provides a solution that we plan to use in similar future studies. It is likely that in most similar targeted NGS studies, the site and variant specific technical error will range over more than two orders of magnitude, as observed in this study, and as we previously reported [16].

## Conclusion

Based on evidence presented here, measurement of DNA variants in the 0.05–1.0% VAF range will enable more informative analysis of AEC somatic mutations associated with cancer risk. Among lung cancer subjects, TP53 mutations were more prevalent (*p* < 0.05) and significantly more enriched for tobacco smoke and age signatures compared to non-cancer subjects matched for smoking and age.

## Supplementary information

**Additional file 1: Table S1.** Determination of Cancer-Free Status in Non-Cancer Subjects.

**Additional file 2: Table S2.** Target-Specific Primers.

**Additional file 3: Table S3.** Characteristics of variants identified in normal AEC specimens.

**Additional file 4: Table S4.** Target- and substitution-specific mutations among all 19 subjects. Target- and substitution-specific mutations among all 19 subjects. Mutation number and prevalence for each substitution type with mutation prevalence (mutations/target bp/subject) in parentheses. Mutations were identified as VAF (variant allele reads/total reads) > 5 × 10^− 4^ (0.05%) and significantly above IS background VAF at respective site based on contingency table analysis (see Methods).

**Additional file 5: Fig. S1.** Qiagen CLC Genomics Workbench Settings.

**Additional file 6: Fig. S2.** Missense mutations in hotspot regions. Missense mutations in hotspot regions (see Methods).

**Additional file 7: File 1.** Custom Perl script used to separate NT and IS reads for parallel variant analysis.

**Additional file 8: File 2.** R scripts used for statistical analysis.

## Data Availability

The datasets generated for this study are available from the corresponding author on reasonable request.

## Abbreviations

AEC: Airway Epithelial Cells
CA-SMK: Cancer subjects, smokers
COSMIC: Catalog of Somatic Mutations in Cancer
FASMIC: Functional Annotation of Somatic Mutations in Cancer
FDA: Food and Drug Administration
HUGO: Human Genome Organization
IS: Internal Standard, synthetic DNA
ISM: Internal Standard Mixture
LCRT: Lung Cancer Risk Test
LDCT: Low Dose Computed Tomography
NC-NON: Non-cancer subjects, non-smokers
NC-SMK: Non-cancer subjects, smokers
NC-TOT: Non-cancer subjects, non-smokers + smokers (all non-cancer subjects)
NGS: Next Generation Sequencing
NT: Native Template, from targeted region of specimen DNA
PCR: Polymerase Chain Reaction
SNP: Single Nucleotide Polymorphism
TCGA: The Cancer Genome Atlas
VAF: Variant Allele Frequency
