# Supplementary material for: Technical advance in targeted NGS analysis enables identification of lung cancer risk-associated low frequency TP53, PIK3CA, and BRAF mutations in airway epithelial cells
Source: BMC Cancer. 2019 Nov 11;19:1081. doi: 10.1186/s12885-019-6313-x (PMC6844032; doi:10.1186/s12885-019-6313-x)
Supplement: Supplementary file 5 — Additional file 5: Fig. S1. Qiagen CLC Genomics Workbench Settings. [file 12885_2019_6313_MOESM5_ESM.docx]

**Supplemental Tables and Figure**

**Supplemental Figure 1. Qiagen CLC Genomics Workbench Settings**

QUALITY TRIMMING

Quality trim = Yes

Quality limit = 0.05

Ambiguous trim = Yes

Ambiguous limit = 2

Trim adapter list = Apex Adapter Trim List

Automatic read-through adapter trimming = Yes

Use colorspace = No

Remove 5' terminal nucleotides = No

Remove 3' terminal nucleotides = No

Discard short reads = Yes

Minimum number of nucleotides in reads = 75

Discard long reads = No

Save discarded sequences = No

Save broken pairs = No

SPLITTING

SIST3.1.pl

ALIGNMENT

References = Homo sapiens (hg19) sequence

Masking mode = No masking

Match score = 1

Mismatch cost = 2

Cost of insertions and deletions = Linear gap cost

Insertion cost = 3

Deletion cost = 3

Length fraction = 0.75

Similarity fraction = 0.9

Global alignment = No

Auto-detect paired distances = Yes

Non-specific match handling = Ignore

Output mode = Create stand-alone read mappings

Create report = Yes

Collect un-mapped reads = No

LOCAL REALIGNMENT

Realign unaligned ends = Yes

Multi-pass realignment = 3

Guidance-variant track = Not set

Maximum guidance-variant length = 200

Output mode = Create stand-alone read mappings

Output track of realigned regions = No

BASIC VARIANT DETECTION

Ploidy = 2

Ignore positions with coverage above = 50,000,000

Restrict calling to target regions =

SEQC23_amplicons_primers_removed_BED

Ignore broken pairs = No

Ignore non-specific matches = Reads

Minimum coverage = 10,000

Minimum count = 5 for NT, 1 for IS

Minimum frequency (%) = 0.001

Base quality filter = Yes

Neighborhood radius = 5

Minimum central quality = 20

Minimum neighborhood quality = 15

Read direction filter = No

Relative read direction filter = No

Read position filter = No

Remove pyro-error variants = No

Create track = Yes

Create annotated table = Yes
