# Supplementary figures and images for: Technical advance in targeted NGS analysis enables identification of lung cancer risk-associated low frequency TP53, PIK3CA, and BRAF mutations in airway epithelial cells
Source: BMC Cancer. 2019 Nov 11;19:1081. doi: 10.1186/s12885-019-6313-x (PMC6844032; doi:10.1186/s12885-019-6313-x)

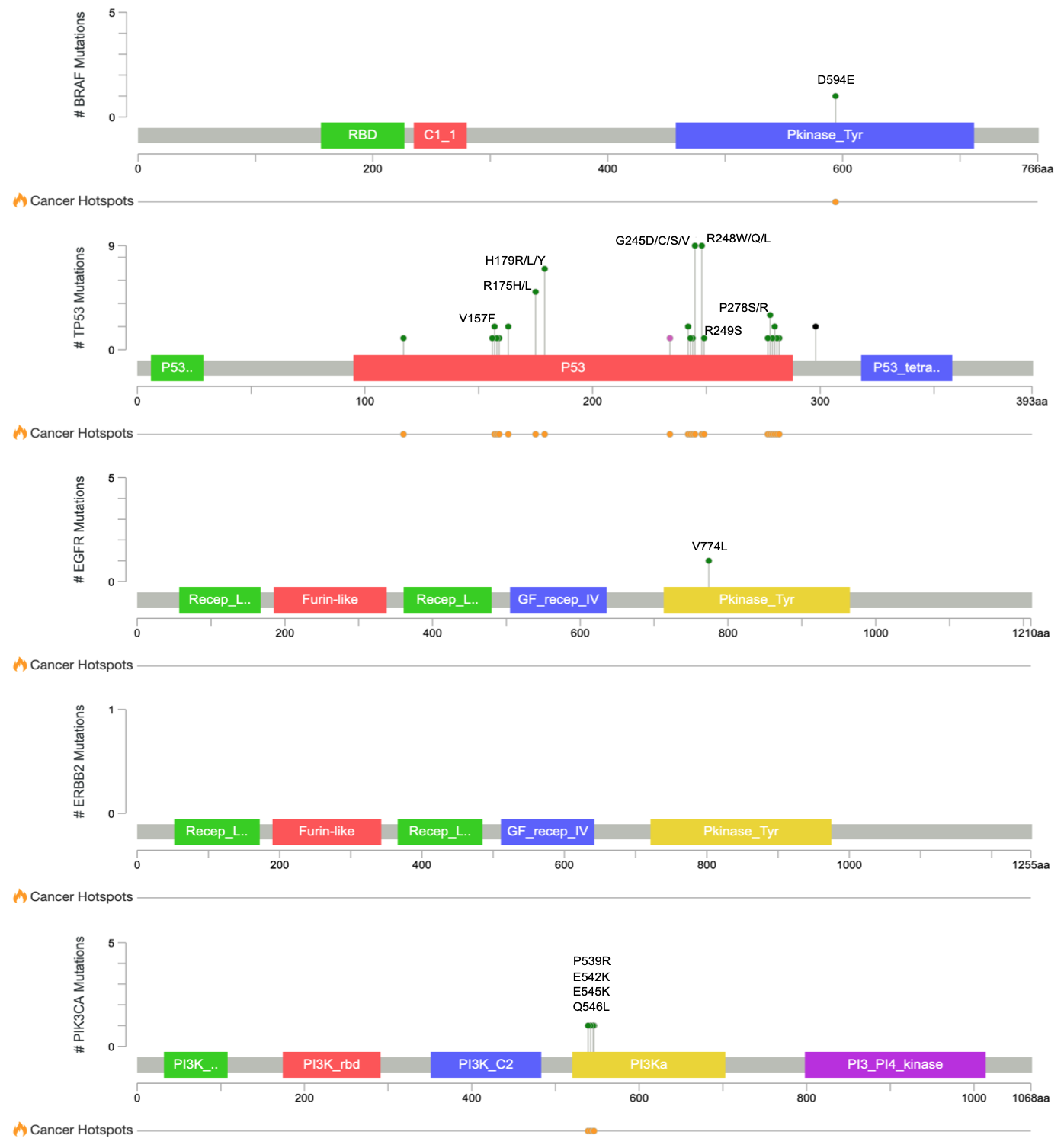

Supplement: Supplementary file 6 — Additional file 6: Fig. S2. Missense mutations in hotspot regions. Missense mutations in hotspot regions (see Methods). [file 12885_2019_6313_MOESM6_ESM.png]
