# Supplementary material for: Technical advance in targeted NGS analysis enables identification of lung cancer risk-associated low frequency TP53, PIK3CA, and BRAF mutations in airway epithelial cells
Source: BMC Cancer. 2019 Nov 11;19:1081. doi: 10.1186/s12885-019-6313-x (PMC6844032; doi:10.1186/s12885-019-6313-x)
Supplement: Supplementary file 7 — Additional file 7: File 1. Custom Perl script used to separate NT and IS reads for parallel variant analysis. [file 12885_2019_6313_MOESM7_ESM.pdf]

```
####################
# SIST: Separate Accugenomics Spike-In reads
# Version 3.1x
#
# Introduction
# Spike-In Separation Toolbox (SIST) is developed for separate Accugenomics sequencing spike-
# in controls (IS reads) from original sample (NT reads).
# The separation is based on the unique characteristics of spike-in controls, which contains
#several di-nucleotides inside the target region.
#
# NOTE: SIST produces IS and NT sequence read files.  Downstream pipeline analysis should use
# the control reference file (e.g., hg19_IS) when
# processing IS files and regular native reference genome when processing NT files.
#
# This toolbox has been specifically designed and used in Sequencing Quality Control 2 project -
#Working Group II
# for the purpose of target sequencing quality control. To read more details about SEQC2
#project, please visit
#
#https://www.fda.gov/ScienceResearch/BioinformaticsTools/MicroarrayQualityControlProject/
#ucm507935.htm
#
# This script is based on SIST3.0, created by Leihong Wu (Leihong.Wu@fda.hhs.gov)
#
# #### Updates: ####
# 04/11/2019: Added ability to use file or paths with space in name.
#           Reduced score for bases with low qscore.
#           Provide 'strict=true' option to partition IS and NT recombinant reads into SUS bin.
#           Until more detailed study is performed, recommend using regular reference genome
#(e.g., hg19), and create control reference genome substituting control bases (e.g., hg19_IS).#
#12/12/2018: Major change: examine just DN bases to sequences aligned ISREF or NTREF, then
#pick best fit for sorting, equal DN IS = NT count or no DN
# are suspicious.
# 09/14/2018: fix some bugs and adding error message of missing picard Ver. 3.01)
# 09/12/2018: Major Change: (three bins stratey) adding one category called "suspected reads"
#which includes low quality reads. (forked version: Ver. 3.00)
#                               the version jumps to 3.00 in order to the three bins strategy. the two
#version will update separately.
# 08/16/2018: using git-hub for version control (update information depreciated)
# 08/02/2018: provide an option to use customized spike-in reference. (Ver. 1.10 -> 1.20)
# 06/09/2018: Support BAM output (to include additional read information)
# 01/04/2018: Support BAM input (Ver. 1.01 -> 1.10)
# 12/02/2017: Fix a Bug about read head detection in FASTQ file generation step. (ver. 1.00 ->
#1.01)
```

```
##### End of Updates#####
#
# Contact tmorrison@accugenmics.com for further assistance.
# Thanks to Leihong Wu (leihong.wu@fda.hhs.gov) for writing the I/O routines and formulating
# the initial splitting approach.
############################################################

use Getopt::Long;
use strict;
use warnings;

############################################################
# USAGE
my $USAGE =<<USAGE;
   Usage:
     SIST3.pl [-fastq_1=<fastq.gz> -fastq_2=<fastq.gz>] [-bam=<bam>] -O=<output_prefix>
[options]

   Required inputs:
     -fastq_1  paired-end reads 1 (required if no bam input)
     -fastq_2  paired-end reads 2 (required if paired-end reads)

     -bam  input file as bam format (required if no fastq input)

     -O    Output file header (*.NT* or *.IS* with endings .vcf to indicate differing bases,
                                    .fasta to indicate reference genome,
                                    .region is a fasta >ID<TAB>CONTIG<TAB>START<TAB>STOP of
control region; used to create vcf)

   Common options:
     -ref [prefix]: prefix of spike-in reference, (defalt: "Refs/Accugenomics_Spikein_V2");
     -type [match, all, gzip]
       match: only extract the spike-in reads
       all:   generate fastq file for spike-in reads and remain origin reads (default)
       gzip:  generate gz file instead of fastq file
     -keepBam : when the input is bam file and keepBam is specified, output would keep the sam
format and not change to fastq;

   Other options:
     -t [num]: threads used in BWA mem (default: 8);
     -bwa_b [num]: mismatch penalty used in BWA mem (default: 6);
     -bwa_o [num,num]: indel penalty used in BWA mem (default: [20,20]);
     -min_length [num]: minimum match length for a read (default: 70);
     -r_hg : human reference genome (default: Refs/genome.fa);
```

```
    -help:  Prints out this helpful message

USAGE
#
########################################################

my $SAMPLE_1 = '';
my $SAMPLE_2 = '';
my $BAM = '';
my $hq_header = '';
my $help='';
my $threads = 4;
my $type = 'all';
my $BWA_B= 4; #6; # penalty for mismatch. default = 4 in BWA MEM for short reads
my $BWA_O= '6,6'; #'20,20';  # 20,20 penalty for indels. default = 6, high value because indels
are highly not expected in spike-in sequence.
my $single_mode = 'false';
my $samtools_path = 'samtools';
my $test_mode = 'false';
my $ref_prefix = 'Refs/ISREF';
my $mismatch = 1; # what level of mismatch are allowed.
my $IS_bed='';

#### TESTING MODE ONLY #####
# my $samtools_path = '/storage2/lwu/SEQC2/samtools-1.8/build/bin/samtools'; # specific
samtools path for test use.
############################

GetOptions (
	"fastq_1=s" => \$SAMPLE_1,
	"fastq1=s"      => \$SAMPLE_1,
	"fq_1=s"        => \$SAMPLE_1,
	"fq1=s"     => \$SAMPLE_1,
	"f1=s"      => \$SAMPLE_1,
	"1=s"       => \$SAMPLE_1,

	"fastq2=s"      => \$SAMPLE_2,
	"fastq_2=s"     => \$SAMPLE_2,
	"fq_2=s"    => \$SAMPLE_2,
	"fq2=s"     => \$SAMPLE_2,
	"f2=s"      => \$SAMPLE_2,
	"2=s"       => \$SAMPLE_2,
```

```
        "strict=s"  => \(my $StrictSus='false'), #set to true if you want recombination reads to go
to SUS bin

    "bam=s"     => \$BAM,

    "O=s"       => \$hq_header,
    "o=s"       => \$hq_header,

    "ref=s"     => \$ref_prefix,
    "r=s"       => \$ref_prefix,
    "R=s"       => \$ref_prefix,
    "Ref=s"     => \$ref_prefix,

                "maxmismatch=i" => \$mismatch,
                "m=i" => \$mismatch,

        "bed=s"     => \$IS_bed,
    "type=s"    => \$type,
    "t=s"       => \$threads,
    "bwa_b=s"   => \$BWA_B,
    "bwa_o=s"   => \$BWA_O,
    q(help)     => \$help,
    q(h)        => \$help,
    q(keepBam)  => \my $keepBam,
                q(umi)              => \my $umi_tag,
    # ONLY USED FOR DEV PURPOSES;
    "test_mode=s"   => \$test_mode
);

## Code Section 1. Pre-Check Begin ##
# c1.1 Help message
if ($help || (!$SAMPLE_1 && !$BAM)) {
   # print "\tSomething Wrong\n";
   print "$USAGE\n";
   exit 0;
}

# c1.2 Check analysis type (-type)
if ($type ne 'match' && $type ne 'all' && $type ne 'gzip' ){
   print('Wrong type! should be one in [match, all, gzip] '."\n");
   exit 0;
}

# c1.3 Check Picard (only works when doing insert size measurement)
```

```
#if (! -f 'Refs/picard.jar' && $eval){
#   print('Missing Picard. create a link of picard  Refs/picard.jar'."\n");
#   exit 0;
#}
#XXXX integrate whole genome controls and don't break the current ref>bed creation.
# c1.5 Check Input Files Availability (-f1, -f2, -bam)
if (-f $BAM) {
        if ($BAM !~ /\.bam$/ ){
        print "Not supported BAM input file format! Must ended with .bam \n";
        exit 1;
        }
        print "Detected uBAM as input\n";
}
elsif (-f $SAMPLE_1 && -f $SAMPLE_2 ) {
        print "Detected READ1 & READ2 FASTQ inputs\n";
}
elsif (-f $SAMPLE_1 ) {
        print "Detected Single Read FASTQ input\n";
        $single_mode = 'true';
}
else {
        print "No input files detected\n";
        exit 1;
}

if ( (! -f ($ref_prefix.'.IS.fasta')) || (! -f ($ref_prefix.'.NT.fasta'))) {
   print " Missing FASTQ reference file(s)!\n";
   print " Use -help to get more information.\n";
   exit 1;
}

if (! -f ($ref_prefix.'.IS.region' || $ref_prefix.'.NT.region')){
   print " Missing Internal Standard region file used to map control coordinates!\n";
   print " Use -help to get more information.\n";
   exit 1;
}


if ($SAMPLE_1){
   if ($SAMPLE_1 !~ /\.fa*s*t*q$/ && $SAMPLE_1 !~ /\.fa*s*t*q\.gz$/){
      print "Not supported Fastq file format for Fastq_1! Must ended with .fastq[.gz] or .fq[.gz]
\n";
      exit 1;
   }
```

```
}

if ($SAMPLE_2){
   if ($SAMPLE_2 !~ /\.fa*s*t*q$/ && $SAMPLE_2 !~ /\.fa*s*t*q\.gz$/){
       print "Not supported Fastq file format for Fastq_2! Must ended with .fastq[.gz] or .fq[.gz]
\n";
       exit 1;
   }
}

# c1.6 Check Output Header
if (!$hq_header){
   print " Missing output folder!\n";
   print " Use -help to get more information.\n";
   exit 1;
}


# c1.8 Create tmp folder if not existed.
my $tmp_dir = 'tmp';
mkdir($tmp_dir) unless(-d $tmp_dir);

## Pre-Check End ##


## Code Section 2. Variable Preparation Begin ##
# c2.1 Create output files
my $hq_file_is  = $hq_header.".IS.txt";
my $hq_file_ntm  = $hq_header.".NTM.txt";
my $hq_file_ntns  = $hq_header.".NTNS.txt";
my $hq_file_ntum  = $hq_header.".NTUM.txt";
my $hq_file_sus = $hq_header.".SUS.txt";
my $sam_file_sus = $hq_header.".SUS.sam";

my $is_fastq_1  = $hq_header.".IS.1.fastq";
my $nt_fastq_1  = $hq_header.".NT.1.fastq";
my $sus_fastq_1 = $hq_header.".SUS.1.fastq";

my $is_fastq_2 = $hq_header.".IS.2.fastq";
my $nt_fastq_2 = $hq_header.".NT.2.fastq";
my $sus_fastq_2 = $hq_header.".SUS.2.fastq";

if ($keepBam){
        $is_fastq_1 = $hq_header.".IS.1.bam" ;
```

```
        $nt_fastq_1 = $hq_header.".NT.1.bam";
        $sus_fastq_1 = $hq_header.".SUS.1.bam";
}

# if output file already exists; overwrite it.
print "Warning: output files (".$hq_header.".*) already exists; will overwrite! \n" if (-f
$hq_file_is);

print("Calculating ISREF scores...\n");
my $seqkeyIS = ID_SEQ('IS','NT');
my %seqkeyIS = %{$seqkeyIS};

print("Calculating NTREF scores...\n");
my $seqkeyNT = ID_SEQ('NT','IS');
my %seqkeyNT = %{$seqkeyNT};

print("Sorting fragments...\n");


my %IS_reads =();
my %NT_reads =();
my %SUS_reads=();

#Sort FASTQ file into native: control: suspect.
#best fit of fragments to ISREF or NTREF (NT + IS counts) because alignment better.
##native #:0:# or #:0:0 or 0:0:0 or 0:0:#
#control 0:#:# or 0:#:0
#suspicious #:#:0, qscore and count of each will resolve this.
#possible improvement: track POS instead +1, allows duplicate removal thereby read overlap
sites don't get double counted.
while(my($key,$scoreIS) = each %seqkeyIS) {
        my $scoreNT='0:0:0';
        my($a1,$b1,$c1)= ($scoreIS =~ /[\.0-9]+/g);
        if (exists ($seqkeyNT{$key})) {$scoreNT = $seqkeyNT{$key};} #Every seqkeyIS but not
seqNT, logic: IS best chance in ISseq, otherwise bin NT
        my($a2,$b2,$c2)= ($scoreNT =~ /[\.0-9]+/g);

        if (($a1 + $b1) > ($a2 + $b2)) {
                if  ($a1 == $b1 || ($StrictSus eq 'true' && $a1 > 1 && $b1 > 1)) {
                        $SUS_reads{$key} = $scoreIS; #can't tell which is dominate
                }elsif ($b1>$a1) {
                        $IS_reads{$key} = $scoreIS; #more control bases than native
                }else {
                        $NT_reads{$key} = $scoreIS; #what ever's left
```

```
                }
                #NOTE IS and NT scores are flipped for seqkeyNT.  Its IS:NT:SUS for seqkeyNT
        } elsif ($a2 == $b2  || ($StrictSus eq 'true' && $a2 > 1 && $b2 > 1)) {
                $SUS_reads{$key} = $scoreNT; #can't tell which is dominate
        } elsif ($a2>$b2) {
                $IS_reads{$key} = $scoreNT; #more control bases than native
        } else {
                $NT_reads{$key} = $scoreNT; #what ever's left
        }
}

## generate fastq files
print("Writing fragement files...\n");
if ($type eq 'all' || $type eq 'gzip'){
        #print "Done. Now generating output fastq/bam files ... \n";
  print "Warning: defined output files already exists; will overwrite! \n" if (-f $is_fastq_1);

  # initialize
  my $is_fastq_1_sam = $is_fastq_1;
  my $nt_fastq_1_sam = $nt_fastq_1;
  my $sus_fastq_1_sam = $sus_fastq_1;

  my $signal = 0;
  my $count_pair_1= 0;
  my $read_line_i = 0;

  # separating fastq file 1
  if($SAMPLE_1){
        #print ('Extracting Reads ... (Pair - 1)'."\n");
                if ($SAMPLE_1 =~ /\.gz$/){
                        open(FH,"gzip -cd \'".$SAMPLE_1."\' |");
                }else{
                        open(FH,"less \'".$SAMPLE_1."\' |");
                }
  }elsif($BAM){
        #print ('Extracting Reads from BAM ... '."\n");
    if ($keepBam){
      open(FH,$samtools_path." view \'".$BAM."\'|");

      $is_fastq_1_sam =~ s/\.bam/\.sam/g;
      $nt_fastq_1_sam =~ s/\.bam/\.sam/g;
      $sus_fastq_1_sam =~ s/\.bam/\.sam/g;

    }else{
```

```
            open(FH,$samtools_path." fastq \'".$BAM."\'|");
        }
    }
    if ($keepBam){
        open(OFH_spike,'>'.$is_fastq_1_sam);
        open(OFH_origin,'>'.$nt_fastq_1_sam);
        open(OFH_suspect,'>'.$sus_fastq_1_sam);

        open(FH_header, $samtools_path." view -H \'".$BAM."\'|");
        while(<FH_header>){
            # output original sam header;
            print OFH_spike $_;
            print OFH_origin $_;
            print OFH_suspect $_;
        }
        close FH_header;
    }else{
        open(OFH_spike,'>'.$is_fastq_1);
        open(OFH_origin,'>'.$nt_fastq_1);
        open(OFH_suspect,'>'.$sus_fastq_1);
    }

            my $array = "";
    while(<FH>){
        #read data from the original fastq/bam files.
        my $line = $_;
        if($keepBam){
            my @array = split("\t",$line);
                    if (exists $IS_reads{$array[0]}) {
                            print OFH_spike $line;
                    }elsif (exists $NT_reads{$array[0]}) {
                            print OFH_origin $line;
                    }elsif (exists $SUS_reads{$array[0]}) {
                            print OFH_suspect $line;
                    }else {
                            print OFH_origin $line; #when not mapped to control regions, does not
exist
                    }
        }else{
            if ($read_line_i % 4 ==0){ # only test on the head line
                    # Casava 1.8 format || Illumina reads ends with /1 or /2
                    if (/^@(\S+)\s(\S+)/ || /^@(\S+)\/[12]$/){
                            $array=$1;
                    }elsif(/^@(.+)/){
```

```
                        $array=$1;
                        chomp($array);
                }
        }
        $read_line_i = $read_line_i + 1 ;
        if (exists $IS_reads{$array}) {
                print OFH_spike $line;
        }elsif (exists $NT_reads{$array}) {
                print OFH_origin $line;
        }elsif (exists $SUS_reads{$array}) {
                print OFH_suspect $line;
        }else {
                print OFH_origin $line; #when not mapped to control regions, does not exist
        }

        }
}
close FH;
close OFH_spike;
close OFH_origin;
close OFH_suspect;

# If paired-end reads;
if($SAMPLE_2){
   # separating fastq file 2
        #print ('Extracting Reads... (Pair - 2)'."\n");
   if ($SAMPLE_2 =~ /\.gz$/){
                        open(FH,"gzip -cd \'".$SAMPLE_2."\'|");
                }else{
                        open(FH,"less \'".$SAMPLE_2."\'|");
                }
   open(OFH_spike,'>'.$is_fastq_2)  ;
   open(OFH_origin,'>'.$nt_fastq_2);
   open(OFH_suspect,'>'.$sus_fastq_2);

   $signal = 0;
   $read_line_i = 0;
                my $array = "";
   while(<FH>){
      my $line = $_;
      if ($read_line_i % 4 ==0){ # head line
         # Casava 1.8 format || Illumina reads ends with /1 or /2
         if (/^@(\S+)\s(\S+)/ || /^@(\S+)\/[12]$/){
            $array=$1;
```

```
            }elsif(/^@(.+)/){
                $array=$1;
                chomp($array);
            }
          }
           $read_line_i = $read_line_i + 1 ;
           if (exists $IS_reads{$array}) {
                      print OFH_spike $line;
           }elsif (exists $NT_reads{$array}) {
                      print OFH_origin $line;
           }elsif (exists $SUS_reads{$array}) {
                      print OFH_suspect $line;
           }else {
                      print OFH_origin $line; #when not mapped to control regions, does not exist
           }
       }
       close FH;
       close OFH_spike;
       close OFH_origin;
       close OFH_suspect;
    }

    if($keepBam){
       # Convert sam to bam file.
       system($samtools_path." view -b \'".$is_fastq_1_sam."\' >".$is_fastq_1);
       system($samtools_path." view -b \'".$nt_fastq_1_sam."\' >".$nt_fastq_1);
       system($samtools_path." view -b \'".$sus_fastq_1_sam."\' >".$sus_fastq_1);
       # Remove original sam file.
       system("rm -f \'".$is_fastq_1_sam."\'");
       system("rm -f \'".$nt_fastq_1_sam."\'");
       system("rm -f \'".$sus_fastq_1_sam."\'");
    }
}
# End of the processing.}
print ('Done! In total '.scalar(keys %IS_reads) ." Spike-in reads have been separated.\n");

######   Generating Gzip files ######
if ($type eq 'gzip'){
    print ('Gzip output files.. (may take long time...)'."\n");
    system("gzip -1 \'".$is_fastq_1."\'");
    system("gzip -1 \'".$nt_fastq_1."\'");
    system("gzip -1 \'".$sus_fastq_1."\'");
    if($SAMPLE_2){
       system("gzip -1 \'".$is_fastq_2."\'");
```

```
    system("gzip -1 \'".$nt_fastq_2."\.");
    system("gzip -1 \'".$sus_fastq_2."\.");
  }
  print ('Program finished normally.'."\n");
}

## Main Program End

sub ID_SEQ{
      my ($r1,$r2) = @_;

      # Accugenomics spike-in Reference

      my $REF_IS = $ref_prefix.'.'.$r1.'.fasta';
      my $mut_REF =$ref_prefix.'.'.$r2.'.vcf';

      if (! -f $mut_REF){ #check for special VCF file pointing to just control change position
        prepare_ref($ref_prefix.'.'.$r2);
      }
    if (! -f ($REF_IS.'.sa')){ #check for one of the bwa index created files
      prepare_ref($ref_prefix.'.'.$r1);
    }

      my %muts=();
      open(FH,$mut_REF);
      while(<FH>){
        chomp;
        my @array=split("\t");
        $muts{$array[0].":".$array[1]}=$array[2];
      }
      close FH;

    my %isb; #generate hash table of control positions
    open(FH,$ref_prefix.'.'.$r2.'.region');
    while(<FH>){
      my $line=$_;
        if (/^>\S+/) {
              chomp($line);
      my @array=split("\t",$line);
      @isb{ map { $array[1].":" . $_ } ($array[2]..$array[3])} =map{1}($array[2]..$array[3]);
     }
      }
    close FH;
      #print STDERR "$_ $isb{$_}\n" for (keys %isb);
```

```
## Variable Preparation End ##

## Code Section 3. Main Program Begin ##
###### c3.1: detect Spike-in Reads ######
## c3.1.1 initialize

# output same file
#open(OFH_SAM_SUS,'>'.$sam_file_sus);

# c3.1.2 Find all mapped reads (candidate spike-in reads) from original read set.
if ($SAMPLE_1){
   if ($single_mode eq 'false'){
      # paired-end reads
      open(FH,"bwa mem -h 1 -v 0 -B \'".$BWA_B."\' -O \'".$BWA_O."\' -t ".$threads.'
'.$REF_IS." \'".
         $SAMPLE_1."\' \'".$SAMPLE_2."\' 2>tmp/bwa_run.log|");
          }else{
      # single-end reads
      open(FH,'bwa mem -v 0 -B '.$BWA_B.' -O '.$BWA_O.' -t '.$threads.' '.$REF_IS." \'".
         $SAMPLE_1."\' 2>tmp/bwa_run.log|");
   }
}elsif($BAM){
   # input is Bam
   open(FH,$samtools_path.' fastq '.$BAM.'|bwa mem -p -v 0 -B '.$BWA_B.' -O
'.$BWA_O.' -t '.$threads.' '.$REF_IS.
         ' - 2>>tmp/bwa_run.log|');
}else{
   print("No output option selected\n");
   exit 0;
}

# read output from BWA alignment
#open(FH, '/NGS/DN_test.NT.bam');

my $test=10;
my %seqkey;
while(<FH>){
         if(/^@/){
                  # print header information to sam file
                  #print OFH_SAM_SUS $_;
         }else{
                  my $line=$_;
```

```
			chomp($line);
			my @array = split(/\t/,$line);
			if ($array[2] ne '*' && $line =~ /\tMD:Z:[\d\^ACGT]+\t/ &&
				(exists ($isb{$array[2].':'.$array[3]}) ||
				exists ($isb{$array[2].':'.($array[3]+length($array[9]))}))){
				# Pre-Step : reads preparation
				my $cigar = $array[5];
				my $mdtag = $1 if $line =~ (/\tMD:Z:([\d\^ACGT]+)\t/);
				my $seq = $array[9];
				my $qs = $array[10];
				#put loop to modify $seq to remove number of bases
corresponding to #S and #I (hard clips should be gone already?)
				#Remove all changes that alter the reference base positions.  This
will simplify  determining if control base is present
				my $i = 0;
				my @cigar_arr = ($cigar =~ /\d+[IDSMH]/g);
				foreach (@cigar_arr) {
					if (/[MX=]$/) {$i+=substr($_,0,length($_)-1);}
					elsif (/[ISP]$/) {
						substr($seq,$i,substr($_,0,length($_)-1))="";
						substr($qs,$i,substr($_,0,length($_)-1))="";
					}
					elsif (/[DN]$/) {
						substr($seq,$i,0)="." x substr($_,0,length($_)-1);
						substr($qs,$i,0)="." x substr($_,0,length($_)-1);
						$i+=substr($_,0,length($_)-1);
					}
				}

				#create a hash of mismatches
				my %seqErr;
				$i=-1;
				while ($mdtag =~ /([0-9]+|[GATC])/g) {
					if ($1 =~ /[GATC]/) {
						++$i;
						$seqErr{$array[2].":".($array[3] + $i)} =
substr($seq,$i,1);
					} else {
						$i+=$1;
					}
				}
				#create hash for each base of seq
				my %mseq;
				my %qseq;
```

```
                        $i=-1;
                        while ($seq =~ /./g) {
                                ++$i;
                                $mseq{$array[2].":".($array[3] + $i)} = substr($seq,$i,1);
                                $qseq{$array[2].":".($array[3] + $i)} = substr($qs,$i,1);
                        }

                        #sort through each control base pos and count
                        my $nativeCount=0;
                        my $controlCount=0;
                        my $susCount=0;
                        while(my($key,$base) = each %mseq) {
                                if (exists $muts{$key}) {
                                        #if ($array[0] eq
"NS500613:182:HNJL5BGX5:2:23110:9141:1367"){
                                        #       print("Current base: ".$key.":".$base."\n");
                        #       if (exists($seqErr{$key})){print("ALT:
".$seqErr{$key}."\n")}else{print("Matches REF\n")};
                        #       print("VCF: ".$muts{$key}."\n");
                                        #       print("Qscore:
".$qseq{$key}."\t".(ord($qseq{$key})-33)."\n");
                        #}
                                        if (not (exists $seqErr{$key})) { #control position
but matches reference
                                                if (ord($qseq{$key}) - 33 < 21)
{$controlCount+=0.5;} else {++$controlCount;}
                                        } elsif ($muts{$key} eq $base) { #control position
matches other reference
                                                if (ord($qseq{$key})-33 < 21 )
{$nativeCount+=0.5;} else {++$nativeCount;}
                                        } else {
                                                if (ord($qseq{$key})-33 < 21)
{++$susCount;} else {++$susCount;} #neither control or genome ref
                                        }
                                }
                        }
                        #generate native/control/sus score for each read pair
                        if (exists $seqkey{$array[0]}){
                                my($a,$b,$c)= ($seqkey{$array[0]}=~/[\.0-9]+/g);

        $seqkey{$array[0]}=($a+$nativeCount).":".($b+$controlCount).":".($c+$susCount);
                        }else{

        $seqkey{$array[0]}=$nativeCount.":".$controlCount.":".$susCount;
```

```
					}
					#if ($array[0] eq
"NS500613:182:HNJL5BGX5:2:23110:9141:1367"){
					#	print("\n".$array[0]."\n");
					#	print ($array[9]."\n");
					#	print ($seq."\n");
					#	print ("cigar: ".$cigar."\n");
					#	print ("mdtag: ".$mdtag."\n");
					#	print("BASE RESULTS: ".$seqkey{$array[0]}."\n");
					#}

				}else{
					#do nothing
				}
			}
		}
		close FH;
		return \%seqkey;
}



sub prepare_ref {
   my ($ref_prefix) = @_;
		print ("customized spike-in reference are used. (".$ref_prefix.") Check required files ...
\n");
   # Create the $mut_ref file of new spike-in reference if not existed.
   my $mut_REF=$ref_prefix.'.vcf';
   my $region_REF=$ref_prefix.'.region';

   if (! -f $mut_REF){
      print ("No Mutation position file detected, try generating them automatically... \n");

		open(FH,$region_REF);
      open(OFH,'>', $mut_REF);
		# Initialize
      my $curr_gene_name = '';
      my $i = 0;
      my @array1;
      while ( <FH> ) {
         my $line = $_;
            if (/^>\S+/){
           # reset position to 0 when a new gene/chrom started.
           chomp($line);
```

```
                    @array1=split(/\t/,$line);
            $i = 0;
             }else {
            my $string = $_;
            chomp($string);
                    foreach (split //, $string){
               ++$i;
               if ($_ =~ /[agct]/){
                              print OFH $array1[1]."\t".($i + $array1[2])."\t${\uc$_}\n";
               }
            }
             }
      }

      close FH;
      close OFH;


      if (-f $mut_REF){
         print("Success.\n");
      }else{
         print("Failed... please contact the developer.\n");
         exit 0;
      }
   }else{
      print ("Mutation position file found!\n");
   }

          # Create BWA INDEX if not existed.
   if (! -f $ref_prefix.'.fasta.bwt'){
      print ("BWA index files are not detected, try generating them automatically... \n");
      system('bwa index '.$ref_prefix.'.fasta ');
      if (-f $ref_prefix.'.fasta.bwt'){
         print ("Successfully generate BWA index. \n");
      }else{
         print ("Failed, please check BWA availability ... \n");
         exit 0;
      }
   }else{
      print ("BWA INDEX found!\n");
   }

   return;
}
```
